# Intra­molecular 1,5-S⋯N σ-hole inter­action in (*E*)-*N*′-(pyridin-4-yl­methyl­idene)thio­phene-2-carbohydrazide

**DOI:** 10.1107/S2056989020003011

**Published:** 2020-03-17

**Authors:** Valeri V. Mossine, Steven P. Kelley, Thomas P. Mawhinney

**Affiliations:** a Department of Biochemistry, University of Missouri, Columbia, MO 65211, USA; b Department of Chemistry, University of Missouri, Columbia, MO 65211, USA

**Keywords:** crystal structure, 4-pyridine­carboxaldehyde 2-thienyl hydrazone, chalcogen bonding, hydrogen bonding, Hirshfeld surface, inter­molecular inter­action energies, energy frameworks

## Abstract

The title compound, C_11_H_9_N_3_OS, (**I**), crystallizes in the monoclinic space group *P*2_1_/*n*. The mol­ecular conformation is nearly planar and features an intra­molecular chalcogen bond between the thio­phene S and the imine N atoms. Within the crystal, the strongest inter­actions between mol­ecules are the N—H⋯O hydrogen bonds, which organize them into inversion dimers. The dimers are linked through short C—H⋯N contacts and are stacked into layers propagating in the (001) plane. The crystal structure features π–π stacking between the pyridine aromatic ring and the azomethine double bond. The calculated energies of pairwise inter­molecular inter­actions within the stacks are considerably larger than those found for the inter­actions between the layers.

## Abstract 2

The hydrazide-hydrazone forms inverse dimers *via* hydrogen bonding, but its conformation is defined by the presence of an intra­molecular chalcogen bond. Electrostatic forces dominate in the crystal packing and give rise to a layered supra­molecular structure.

## Chemical context   

Hydrazones are a versatile group of organic structures that have been the subject of numerous studies in chemical (Barluenga & Valdés, 2011 ▸), biomedical (Narang *et al.*, 2012 ▸), and materials (Serbutoviez *et al.*, 1995 ▸) sciences for decades. For example, hydrazone-based iron chelators have found applications as analytical reagents (Singh *et al.*, 1982 ▸) and have been proposed for the treatment of bacterial, fungal, and protozoan infections (Narang *et al.*, 2012 ▸; Rzhepishevska *et al.*, 2014 ▸), as well as health disorders involving alterations in iron metabolism, such as hemochromatosis (Jansová & Šimůnek, 2019 ▸), cancer (Lovejoy & Richardson, 2003 ▸), and neurodegenerative diseases (Richardson, 2004 ▸). In addition, since iron has been identified as a critical co-factor of bacterial phenazine cytotoxicity to mammalian host cells (Mossine *et al.*, 2016 ▸), the application of efficient iron chelators to the infection sites could not only restrict proliferation of the pathogen but also protect the infected tissue from injury caused by toxic bacterial metabolites (Mossine *et al.*, 2018 ▸).
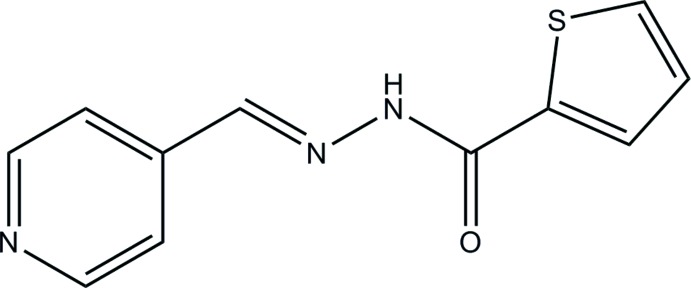


As a part of our search for potent inhibitors of cytotoxic virulence factors from drug-resistant *Pseudomonas aeruginosa*, we have prepared 4-pyridine­carboxaldehyde 2-thienyl hydrazone (**I**), a structural analog of a series of hydrazide-hydrazones that have proved to be pharmacologically active *in vivo*. Here we report on the mol­ecular and crystal structures of (**I**), with an emphasis on the non-covalent inter­actions in the structure.

## Structural commentary   

**Figure 1:**
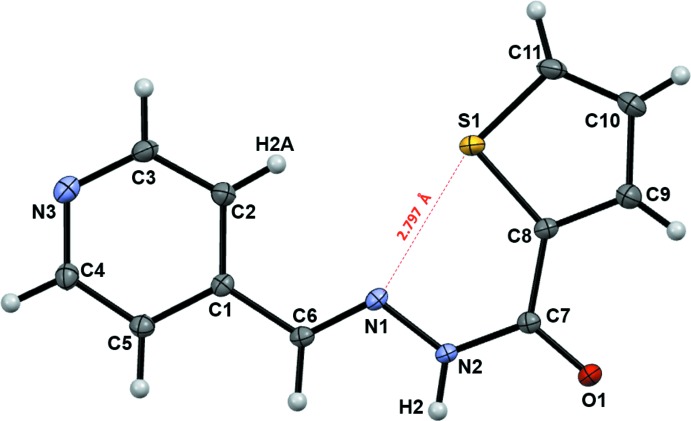
Atomic numbering and displacement ellipsoids at the 50% probability level for (**I**). The intra­molecular chalcogen bond is shown as a dotted line.

**Figure 2:**
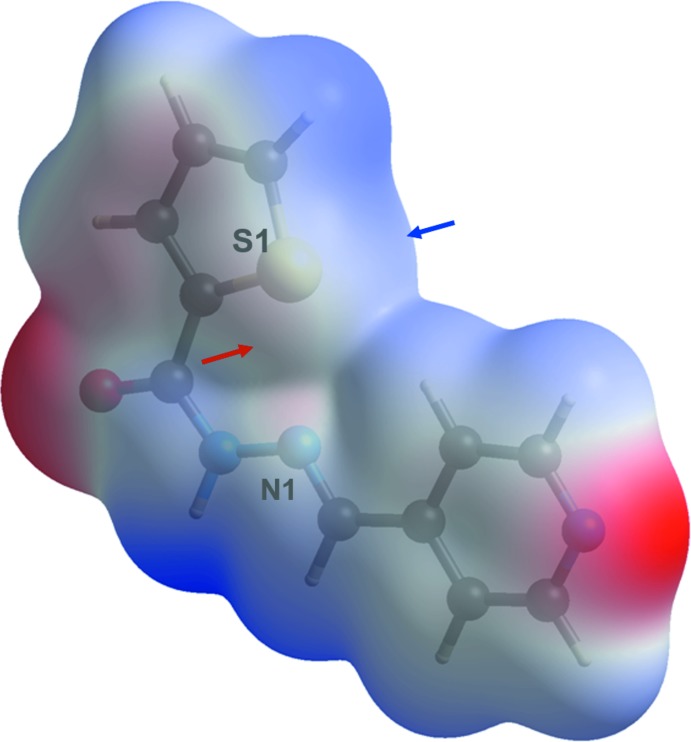
Electrostatic potential mapped on the promolecule 0.002 a.u. isosurface of (**I**), in the range −0.0750 to +0.0806 a.u., red indicates regions of negative ESP and blue indicates regions of positive ESP. The red arrow points at the region of negative ESP that is consistent with topography of aromatic *p*-electrons originating from the S1 atom and directed *away* from the mol­ecular plane. The blue arrow points at the region of positive ESP associated with electronegative S1 (σ-hole) and located *within* the mol­ecular plane. Calculations were done using a 6–311 G(d,p) basis set at the B3LYP level of theory.

The mol­ecular structure and atomic numbering are shown in Fig. 1 ▸. The mol­ecule is essentially flat, with exception of the H2 and the thio­phene ring carbon and hydrogen atoms, which deviate from the mol­ecular plane by more than 0.1 Å; the dihedral angle between the planes formed by the pyridine and the thio­phene rings is only 8.28 (7)°. The configuration around the azomethine N1—C6 bond is *trans* with respect to C2 and N1, as would be expected for the structure. The conformation around the N2—C7 bond, with respect to the N1 and C8 atoms, is *cis*, however. Such a *syn*-periplanar conformation is unusual for aromatic hydrazide-hydrazones and indicates the presence of additional intra­molecular inter­actions that could stabilize the energetically unfavorable arrangement around the amide bond. Specifically, the inter­atomic S1⋯N1 distance is 2.7971 (11) Å, which is shorter than the sum of the van der Waals radii by 0.55 Å (Table 1 ▸), thus indicating the presence of a chalcogen bond (Scilabra *et al.*, 2019 ▸). In addition, other geometric features of the mol­ecule are in concord with the definition (Aakeroy *et al.*, 2019 ▸) of the bond. The angle between the S1—C11 σ covalent bond and the S1⋯N1 suspect is 164.17 (5)°, which makes the latter an extension of the former. The S1⋯N1—C6 angle is 148.48 (8)°, the S1 donor is in the mol­ecular plane and approaches the N1 acceptor roughly along the axis of the lone pair. In addition, a comparison of the bond lengths in (**I**) and its structural analogues, 2-thio­phene carb­oxy­lic acid (Tiekink, 1989 ▸), 2-thio­phene carboxamide (Low *et al.*, 2009 ▸), or 4-hy­droxy­benzaldehyde 2-thienylhydrazone (Li *et al.*, 2010 ▸), which lack attractive non-covalent inter­actions at the sulfur atom, revealed that the S1—C11 bond in (**I**) is longer than similar bonds in the reference mol­ecules, by 0.01–0.02 Å. The chalcogen bond is believed to originate from attractive electrostatic inter­actions between regions of positive ESP of a donor, such as the S1 atom, and a lone pair (or a π region) of the acceptor, such as the N1 atom in (**I**). In thio­phene, two *p*-electrons of the sulfur atom participate in aromatic π-bonding, while another lone pair of *p*-electrons occupies the *sp* ^2^ orbital, with the maximum of the electron density localized in the thio­phene ring plane. Nevertheless, there are regions of positive electrostatic potential, conventionally named σ-holes (Scilabra *et al.*, 2019 ▸), which are located opposite to the S—C covalent bonds. For illustrative purposes, we have calculated a distribution of the electrostatic potential over the promolecule isosurface of (**I**), which is shown in Fig. 2 ▸ and which exhibits one of the two σ-holes mapped to the surface.

## Supra­molecular features   

**Figure 3:**
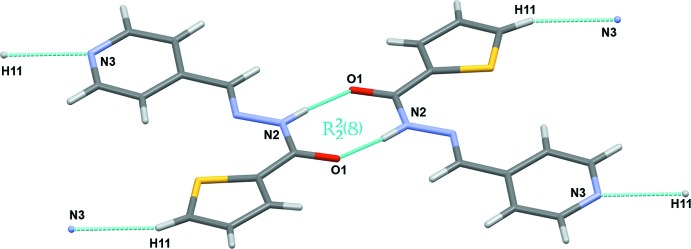
Hydrogen-bonded dimerization of (**I**).

The title compound crystallizes in the monoclinic *P*2_1_/*n* space group, with four equivalent mol­ecules per unit cell. The mol­ecules are organized pairwise as flat dimers (Fig. 3 ▸), with two hydrogen bonds of the same N2—H2⋯O1 type (Table 1 ▸), which are responsible for ‘holding’ the dimers together. This hydrogen-bonding arrangement can be described in terms of the graph-set descriptor 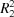
(8). An additional pair of the short C11—H11⋯N3 contacts links the dimers into mol­ecular sheets propagating in the [110] and [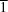 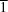
0] directions. The inter­molecular contacts also include the π–π stacking between the pyridine aromatic ring and the azomethine double bond.

**Figure 4:**
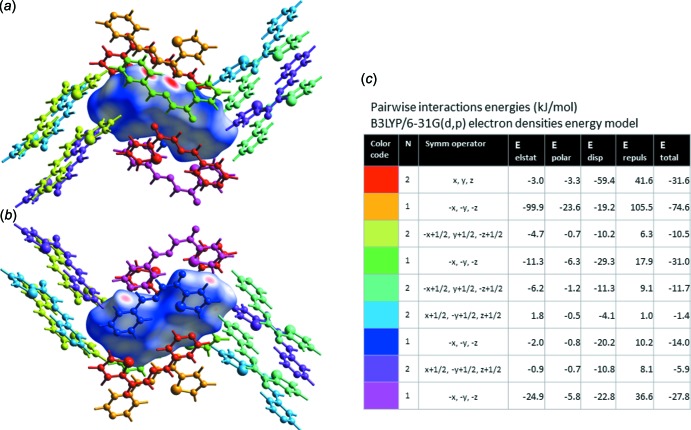
Inter­action energies in crystal structure of (**I**). (*a*), (*b*) Views of inter­actions between a central mol­ecule, shown as its Hirshfeld surface, and 14 mol­ecules that share the inter­action surfaces with the central mol­ecule. (*c*) Calculated energies (electrostatic, polarization, dispersion, repulsion, and total) of pairwise inter­actions between the central mol­ecule and those indicated by respective colors.

**Figure 5:**
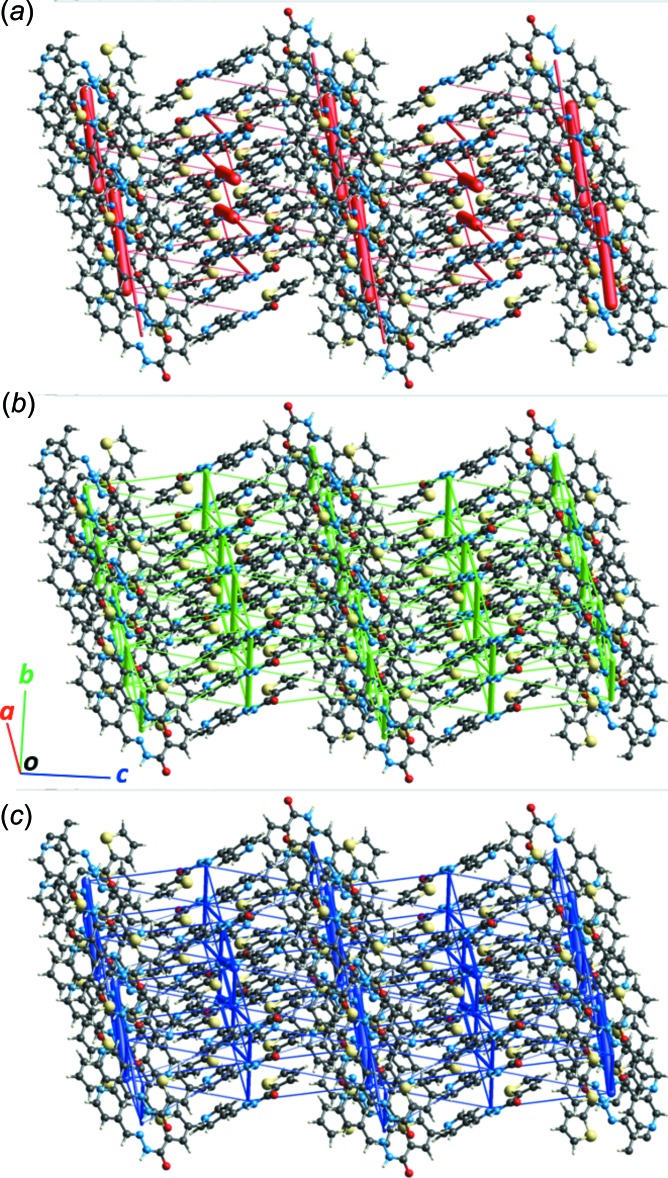
Energy frameworks for separate (*a*) electrostatic and (*b*) dispersion contributions to the (*c*) total pairwise inter­action energies. The cylinders link mol­ecular centroids, and the cylinder thickness is proportional to the magnitude of the energies (see Fig. 4 ▸). For clarity, the cylinders corresponding to energies <5 kJ mol^−1^ are not shown. The directionality of the crystallographic axes is the same for all three diagrams.

To evaluate the contributions of these and other inter­molecular contacts to the energetics of the crystal lattice in (**I**), we calculated pairwise inter­action energies for all unique contacts found in the crystal structure. The results are shown in Fig. 4 ▸. It follows from these data that electrostatic inter­actions within the dimers are the major contributors to the packing forces in the crystal of (**I**). The *Crystal Explorer* software (Spackman *et al.*, 2008 ▸) provides a tool to illustrate the magnitude and directionality of the major inter­actions within a crystal structure, the energy frameworks builder (Turner *et al.*, 2015 ▸). Using the pairwise inter­action energies calculated for (**I**), we obtained energy framework diagrams for the contributions of electrostatic and dispersion forces, as well as for the total energy. The diagrams and crystal packing are shown in Fig. 5 ▸. According to the diagrams, the main crystal packing forces are those that form sheets of the dimers, as well as stacks of the sheets. These stacks are organized in layers that are about 10 Å thick and run in parallel to (001). Inter­molecular contacts between mol­ecules located in neighboring layers are weak.

## Database survey   

The crystal structures of three metal complexes, containing 4-pyridine­carboxaldehyde 2-thienylhydrazone coordinated to copper(I) tri­phenyl­phosphinate, have been published [CSD (Groom *et al.*, 2016 ▸) refcodes CCDC 1401340, 1433202, 1433203; Gholivand *et al.*, 2016 ▸], but the crystal structure of the title compound as a single mol­ecule has not been reported previously. In the same paper, the copper complexes of 2-pyridine­carboxaldehyde 2-thienylhydrazone (CCDC 1433200) and 3-pyridine­carboxaldehyde 2-thienylhydrazone (CCDC 1433201) were also reported. In four complexes, mol­ecules of 3- or 4-pyridine­carboxaldehyde 2-thienylhydrazone act as monodentate ligands bound to the copper ion through the pyridine nitro­gens, are not ionized and do assume conformations close to that of free (**I**), thus suggesting that the intra­molecular chalcogen bonding is retained if coordination to the metal occurs *via* a remote part of the mol­ecule. In contrast, 2-pyridine­carboxaldehyde 2-thienylhydrazone was found to chelate Cu^+^ through the pyridine and the imine nitro­gen atoms, so that the chalcogen bonding between the thio­phene sulfur and the imine nitro­gen atoms was disabled. The dimer-forming hydrogen bonding did survive in the CCDC 1433201, 1433202 and 1433203 structures as well. Not only a coordinated metal ion, such as the aforementioned copper in CCDC 1433200, but also an opportunistic hydrogen bonding can disable the chalcogen bonding in 2-thio­phene­carb­oxy­lic acid-derived hydrazide-hydrazones. For instance, crystalline Schiff bases of 2-thio­phene­carb­oxy­lic acid hydrazide and 4-meth­oxy­benzaldehyde (Li & Jian, 2010 ▸), or 2-acetyl­pyridine (Christidis *et al.*, 1995 ▸) adopt conformations similar to (**I**), thus suggesting a general trend of 1,5-S⋯N chalcogen-bond formation in structures analogous to (**I**). In contrast, in hydrazones formed by condensation of 2-thio­phene­carb­oxy­lic acid hydrazide and 4-hy­droxy­benzaldehyde (Li *et al.*, 2010 ▸) or 2-hy­droxy­aceto­phenone (Jiang, 2011 ▸; Singh *et al.*, 2013 ▸), which have an additional hydrogen-bonding inter­action between the aromatic hydroxyl groups and the imine nitro­gen, the intra­molecular chalcogen bonding is switched to a weaker 1,4-S⋯O_carbon­yl_ contact.

## Synthesis and crystallization   

To a suspension of 2-thio­phene­carb­oxy­lic acid hydrazide (711 mg, 5 mmol) in 15 mL of 70% aqueous EtOH were added 0.536 mg (471 µL, 5 mmol) of 4-pyridine­carboxaldehyde, and the reaction mixture was stirred for 2 h at 343 K. The resulting clear solution was brought to 277 K and left for two days to crystallize as colorless needles. Suitable crystals were then selected for subsequent diffraction studies.

## Refinement details   

Crystal data, data collection and structure refinement details are summarized in Table 2 ▸. All H-atom coordinates were refined freely.

## Supplementary Material

Crystal structure: contains datablock(s) I. DOI: 10.1107/S2056989020003011/rz5271sup1.cif

Structure factors: contains datablock(s) I. DOI: 10.1107/S2056989020003011/rz5271Isup2.hkl

Supporting information file. DOI: 10.1107/S2056989020003011/rz5271Isup3.cml

CCDC reference: 1983191

Additional supporting information:  crystallographic information; 3D view; checkCIF report

## Figures and Tables

**Table 1:** Non-covalent heteroatom inter­actions geometry (Å, °)

Hydrogen bonding				
*D*—H⋯*A*	*D*—H	H⋯*A*	*D*⋯*A*	*D*—H⋯*A*
N2—H2⋯O1^i^	0.902 (18)	1.942 (18)	2.8376 (14)	171.8 (18)
C11—H11⋯N3^ii^	0.936 (19)	2.501 (18)	3.3664 (18)	153.9 (14)
Chalcogen bonding				
*R*—*Ch*⋯*A*			*Ch*⋯*A*	*R*—*Ch*⋯*A*
C11—S1⋯N1			2.7971 (11)	164.17 (5)

Symmetry codes: (i) 1 − *x*, −*y*, −*z*; (ii) 2 − *x*, 2 − *y*, −*z*.

**Table 2:** Experimental details

Crystal data	
Chemical formula	C_11_H_9_N_3_OS
*M*_r_	231.27
Crystal system, space group	Monoclinic, *P*2_1_/*n*
Temperature (K)	100
*a*, *b*, *c* (Å)	12.0600 (8), 4.4531 (3), 19.9528 (13)
β (°)	102.228 (2)
*V* (Å^3^)	1047.24 (12)
*Z*	4
Radiation type	Mo *K*α
μ (mm^−1^)	0.29
Crystal size (mm)	0.49 × 0.04 × 0.01

Data collection	
Diffractometer	Bruker APEXII CCD
Absorption correction	Multi-scan (*AXScale*; Bruker, 2016 ▸)
*T*_min_, *T*_max_	0.694, 0.746
No. of measured, independent and observed [*I* > 2σ(*I*)] reflections	25531, 3767, 2821
*R*_int_	0.059
(sin θ/λ)_max_ (Å^−1^)	0.754

Refinement	
*R*[*F* ^2^ > 2σ(*F* ^2^)], *wR*(*F* ^2^), *S*	0.040, 0.101, 1.03
No. of reflections	3767
No. of parameters	172
H-atom treatment	Only H-atom coordinates refined
Δρ_max_, Δρ_min_ (e Å^−3^)	0.44, −0.29

Computer programs: *APEX3* and *SAINT* (Bruker, 2016 ▸), *SHELXS* (Sheldrick, 2008 ▸), *SHELXL* (Sheldrick, 2015 ▸), *OLEX2*(Dolomanov *et al.*, 2009 ▸), *Mercury* (Macrae *et al.*, 2020 ▸) and *publCIF* (Westrip, 2010 ▸).

## supplementary crystallographic information

### Crystal data

**Table:**

C_11_H_9_N_3_OS	*F*(000) = 480
*M**_r_* = 231.27	*D*_x_ = 1.467 Mg m^−^^3^
Monoclinic, *P*2_1_/*n*	Mo *K*α radiation, λ = 0.71073 Å
*a* = 12.0600 (8) Å	Cell parameters from 8101 reflections
*b* = 4.4531 (3) Å	θ = 2.2–32.3°
*c* = 19.9528 (13) Å	µ = 0.29 mm^−^^1^
β = 102.228 (2)°	*T* = 100 K
*V* = 1047.24 (12) Å^3^	Needle, clear colourless
*Z* = 4	0.49 × 0.04 × 0.01 mm

### Data collection

**Table:**

Bruker APEXII CCD diffractometer	3767 independent reflections
Radiation source: Sealed Source Mo with TRIUMPH optics	2821 reflections with *I* > 2σ(*I*)
Graphite monochromator	*R*_int_ = 0.059
ω and phi scans	θ_max_ = 32.4°, θ_min_ = 2.2°
Absorption correction: multi-scan (*AXScale*; Bruker, 2016)	*h* = −18→18
*T*_min_ = 0.694, *T*_max_ = 0.746	*k* = −6→6
25531 measured reflections	*l* = −30→30

### Refinement

**Table:**

Refinement on *F*^2^	0 restraints
Least-squares matrix: full	Hydrogen site location: difference Fourier map
*R*[*F*^2^ > 2σ(*F*^2^)] = 0.040	Only H-atom coordinates refined
*wR*(*F*^2^) = 0.101	*w* = 1/[σ^2^(*F*_o_^2^) + (0.0482*P*)^2^ + 0.4155*P*] where *P* = (*F*_o_^2^ + 2*F*_c_^2^)/3
*S* = 1.03	(Δ/σ)_max_ < 0.001
3767 reflections	Δρ_max_ = 0.44 e Å^−^^3^
172 parameters	Δρ_min_ = −0.28 e Å^−^^3^

### Special details

**Table:**

Geometry. All esds (except the esd in the dihedral angle between two l.s. planes) are estimated using the full covariance matrix. The cell esds are taken into account individually in the estimation of esds in distances, angles and torsion angles; correlations between esds in cell parameters are only used when they are defined by crystal symmetry. An approximate (isotropic) treatment of cell esds is used for estimating esds involving l.s. planes.

### Fractional atomic coordinates and isotropic or equivalent isotropic displacement parameters (Å^2^)

**Table:**

	*x*	*y*	*z*	*U*_iso_*/*U*_eq_	
S1	0.86385 (3)	0.41227 (8)	0.09663 (2)	0.01759 (9)	
O1	0.57985 (7)	−0.0566 (2)	0.08737 (5)	0.01585 (19)	
N1	0.67837 (8)	0.4814 (2)	−0.01319 (5)	0.0121 (2)	
C3	0.86828 (11)	1.1139 (3)	−0.10148 (7)	0.0177 (3)	
C6	0.63662 (10)	0.6002 (3)	−0.07173 (6)	0.0123 (2)	
N2	0.61184 (9)	0.2710 (2)	0.00868 (5)	0.0125 (2)	
C8	0.75495 (10)	0.1986 (3)	0.11541 (6)	0.0128 (2)	
C7	0.64471 (10)	0.1304 (3)	0.06997 (6)	0.0119 (2)	
N3	0.82339 (9)	1.2476 (3)	−0.16131 (6)	0.0170 (2)	
C1	0.70133 (10)	0.8252 (3)	−0.10079 (6)	0.0117 (2)	
C10	0.89639 (12)	0.1627 (4)	0.21440 (7)	0.0214 (3)	
C2	0.81256 (10)	0.9054 (3)	−0.06925 (7)	0.0158 (2)	
C9	0.78539 (11)	0.0796 (3)	0.18034 (7)	0.0191 (3)	
C5	0.65308 (11)	0.9644 (3)	−0.16254 (6)	0.0146 (2)	
C4	0.71673 (11)	1.1716 (3)	−0.19047 (7)	0.0162 (2)	
C11	0.94837 (11)	0.3405 (3)	0.17495 (7)	0.0190 (3)	
H2	0.5467 (15)	0.214 (4)	−0.0197 (9)	0.023*	
H6	0.5592 (15)	0.543 (4)	−0.0981 (9)	0.023*	
H5	0.5756 (15)	0.918 (4)	−0.1848 (9)	0.023*	
H2A	0.8487 (14)	0.813 (4)	−0.0278 (9)	0.023*	
H4	0.6850 (14)	1.273 (4)	−0.2329 (9)	0.023*	
H9	0.7381 (14)	−0.038 (4)	0.1991 (9)	0.023*	
H3	0.9464 (15)	1.168 (4)	−0.0810 (9)	0.023*	
H11	1.0212 (15)	0.424 (4)	0.1851 (9)	0.023*	
H10	0.9262 (15)	0.103 (4)	0.2563 (9)	0.023*	

### Atomic displacement parameters (Å^2^)

**Table:**

	*U*^11^	*U*^22^	*U*^33^	*U*^12^	*U*^13^	*U*^23^
S1	0.01205 (14)	0.02312 (18)	0.01612 (15)	−0.00603 (12)	−0.00037 (11)	0.00258 (13)
O1	0.0135 (4)	0.0181 (5)	0.0154 (4)	−0.0049 (3)	0.0018 (3)	0.0027 (4)
N1	0.0121 (4)	0.0104 (5)	0.0142 (5)	−0.0024 (4)	0.0038 (4)	−0.0003 (4)
C3	0.0130 (5)	0.0181 (6)	0.0217 (6)	−0.0037 (5)	0.0030 (5)	0.0006 (5)
C6	0.0109 (5)	0.0115 (5)	0.0141 (5)	−0.0010 (4)	0.0022 (4)	−0.0012 (5)
N2	0.0106 (4)	0.0129 (5)	0.0131 (5)	−0.0036 (4)	0.0008 (4)	0.0012 (4)
C8	0.0108 (5)	0.0133 (5)	0.0140 (5)	−0.0018 (4)	0.0021 (4)	−0.0006 (5)
C7	0.0112 (5)	0.0114 (6)	0.0130 (5)	0.0001 (4)	0.0025 (4)	−0.0007 (4)
N3	0.0178 (5)	0.0156 (5)	0.0189 (5)	−0.0027 (4)	0.0069 (4)	−0.0006 (4)
C1	0.0131 (5)	0.0100 (5)	0.0127 (5)	−0.0002 (4)	0.0040 (4)	−0.0014 (4)
C10	0.0176 (6)	0.0279 (7)	0.0158 (6)	−0.0019 (5)	−0.0034 (5)	0.0021 (5)
C2	0.0136 (5)	0.0160 (6)	0.0170 (6)	−0.0008 (5)	0.0011 (4)	0.0019 (5)
C9	0.0162 (6)	0.0244 (7)	0.0156 (6)	−0.0033 (5)	0.0010 (5)	0.0023 (5)
C5	0.0150 (5)	0.0146 (6)	0.0139 (5)	−0.0018 (4)	0.0022 (4)	−0.0013 (5)
C4	0.0205 (6)	0.0152 (6)	0.0134 (5)	−0.0017 (5)	0.0043 (5)	0.0005 (5)
C11	0.0118 (5)	0.0246 (7)	0.0183 (6)	−0.0021 (5)	−0.0018 (5)	−0.0017 (5)

### Geometric parameters (Å, º)

**Table:**

S1—C11	1.7055 (14)	C8—C7	1.4736 (16)
S1—C8	1.7259 (12)	N3—C4	1.3381 (17)
O1—C7	1.2410 (15)	C1—C5	1.3918 (17)
N1—C6	1.2839 (16)	C1—C2	1.4019 (17)
N1—N2	1.3643 (14)	C10—C11	1.360 (2)
C3—N3	1.3413 (18)	C10—C9	1.4161 (19)
C3—C2	1.3821 (18)	C10—H10	0.879 (17)
C3—H3	0.974 (17)	C2—H2A	0.944 (17)
C6—C1	1.4631 (17)	C9—H9	0.912 (18)
C6—H6	1.002 (17)	C5—C4	1.3900 (18)
N2—C7	1.3561 (15)	C5—H5	0.968 (17)
N2—H2	0.901 (18)	C4—H4	0.963 (17)
C8—C9	1.3756 (18)	C11—H11	0.936 (18)
			
C11—S1—C8	91.85 (6)	C2—C1—C6	122.44 (11)
C6—N1—N2	115.48 (10)	C11—C10—C9	112.28 (12)
N3—C3—C2	124.61 (12)	C11—C10—H10	125.6 (12)
N3—C3—H3	115.8 (10)	C9—C10—H10	122.2 (12)
C2—C3—H3	119.5 (10)	C3—C2—C1	118.39 (12)
N1—C6—C1	120.21 (11)	C3—C2—H2A	121.5 (10)
N1—C6—H6	121.1 (10)	C1—C2—H2A	120.1 (10)
C1—C6—H6	118.7 (10)	C8—C9—C10	112.84 (12)
C7—N2—N1	121.75 (10)	C8—C9—H9	122.8 (11)
C7—N2—H2	119.0 (11)	C10—C9—H9	124.3 (11)
N1—N2—H2	119.0 (11)	C4—C5—C1	119.19 (12)
C9—C8—C7	121.75 (11)	C4—C5—H5	121.3 (10)
C9—C8—S1	110.68 (9)	C1—C5—H5	119.5 (10)
C7—C8—S1	127.51 (9)	N3—C4—C5	123.72 (12)
O1—C7—N2	118.73 (11)	N3—C4—H4	115.5 (10)
O1—C7—C8	120.41 (11)	C5—C4—H4	120.8 (10)
N2—C7—C8	120.86 (11)	C10—C11—S1	112.35 (10)
C4—N3—C3	116.38 (11)	C10—C11—H11	129.3 (11)
C5—C1—C2	117.70 (11)	S1—C11—H11	118.3 (11)
C5—C1—C6	119.85 (11)		

### Hydrogen-bond geometry (Å, °)

**Table:**

*D*-H···*A*	*D*-H	H···*A*	D···*A*	D-H···*A*
N2-H2···O1^i^	0.902 (18)	1.942 (18)	2.8376 (14)	171.8 (18)
C11-H11···N3^ii^	0.936 (19)	2.501 (18)	3.3664 (18)	153.9 (14)

Symmetry codes: (i) 1-x,-y,-z; (ii) 2-x,2-y,-z
